# Genomic Damage in Endstage Renal Disease—Contribution of Uremic Toxins

**DOI:** 10.3390/toxins2102340

**Published:** 2010-10-11

**Authors:** Nicole Schupp, August Heidland, Helga Stopper

**Affiliations:** 1 Institute of Pharmacology and Toxicology, University of Würzburg, Versbacher Straße 9, 97078 Würzburg, Germany; Email: stopper@toxi.uni-wuerzburg.de; 2 Department of Internal Medicine, University of Würzburg, Josef-Schneider-Straße 2, 97080 Würzburg, Germany; Email: august.heidland@t-online.de

**Keywords:** dialysis, genotoxicity, uremic toxins

## Abstract

Patients with end-stage renal disease (ESRD), whether on conservative, peritoneal or hemodialysis therapy, have elevated genomic damage in peripheral blood lymphocytes and an increased cancer incidence, especially of the kidney. The damage is possibly due to accumulation of uremic toxins like advanced glycation endproducts or homocysteine. However, other endogenous substances with genotoxic properties, which are increased in ESRD, could be involved, such as the blood pressure regulating hormones angiotensin II and aldosterone or the inflammatory cytokine TNF-α. This review provides an overview of genomic damage observed in ESRD patients, focuses on possible underlying causes and shows modulations of the damage by modern dialysis strategies and vitamin supplementation.

## 1. Introduction

In the United States of America, 13.1% of the population over 20 years were estimated to be suffering from chronic kidney disease in 2004, and in 2007, 0.18% received renal replacement therapy ([1] and United States Renal Data System, www.usrds.org). An approximate 45% increase in prevalence is expected in 2020 due to the increasing age of the population (United States Renal Data System, www.usrds.org). Similar values have been observed in other industrialized nations. Thus, an increasing number of people will receive renal replacement therapy for lengthier periods of time.

Besides causing cardiovascular and other complications, end-stage renal disease (ESRD) is further characterized by a high incidence of cancer [2,3,4]. Of the cancer types recorded in these epidemiological studies, the risk of kidney cancer is tremendously increased. Depending on the continent, it is three- to ten-times higher than that seen in the control population [2].

Several biological parameters of ESRD patients were measured to determine their burden of genomic damage. Structural DNA damage, like single and double strand breaks and alkali labile sites, detectable with one of the standard genotoxicity assays, the comet assay, was significantly increased in lymphocytes of patients under dialysis [5,6,7]. These types of DNA damage are theoretically repairable. Yet, DNA repair capacity is reduced by prolonged hemodialysis therapy [8]. Unrepaired or improperly repaired DNA lesions may have serious consequences, such as premature ageing [9,10], atherosclerosis [11] and cancer predisposition [12].

Furthermore, unrepairable genomic damage *per se*, such as sister chromatid exchanges and chromosome breaks, were found in lymphocytes of dialysis patients [13,14,15]. In addition to the DNA in the nucleus, the mitochondrial DNA was also shown to be affected: deletions and mutations were observed in mitochondrial DNA of muscle cells and in the easily accessible hair follicles [16,17,18]. Furthermore, the most prominent oxidative DNA lesion, the modified base 7,8-dihydro-8-oxo-guanine (8-oxodG), is increased in ESRD [19]. 8-oxodG has a high mutagenic potential and repair errors or unrepaired 8-oxodGs lead to G:C→T:A transversions, which are often found in genes altered in tumors [20].

In this review, the impact of different dialysis therapies and of supplementation with vitamins on genomic damage of dialysis patients will be discussed. Also, underlying mechanisms of the origin of the increased DNA damage in ESRD will be described.

## 2. Causes of Increased Genomic Damage in ESRD

### 2.1. Genotoxic Uremic Toxins

Several minerals and molecules accumulate in the blood of patients with ESRD, which, if not removed, may lead to death. Besides urea, which is the classical uremic toxin, over 90 other toxins were included in the encyclopedic overview of uremic retention solutes initiated by the European Uremic Toxin Work Group (EUTox). These compounds, excreted by healthy kidneys under normal conditions, are classified as low molecular weight water-soluble compounds, middle molecules and protein-bound compounds [21]. In the seven years since the first review of EUTox, over 25 additional solutes have been identified ([22] and EUTox database: http://www.nephro-leipzig.de/eutoxdb/viewtoxins.php). It is conceivable that not all compounds responsible for the uremic syndrome have yet been discovered.

Genotoxic or mutagenic properties were reported for some of the toxins in the database, e.g., hydroquinone, indoxyl sulfate, leptin, methylglyoxal, *N*-ε-(carboxymethyl)lysine and TNF-α [6,23,24,25,26]. Other compounds, like trihalomethanes and heterocyclic amines, not included in the EUTox database but shown to accumulate in ESRD, are known mutagens [27,28]. These toxins, as well as other, not yet recognized substances can contribute to the genomic damage in ESRD by directly causing DNA damage.

### 2.2. Oxidative Stress

Reactive oxygen species (ROS) perform important physiological functions, for example in immune defense or in cellular signal transduction. However, when present in high concentrations they damage cellular structures, including lipids, membranes, proteins and nucleic acids [29]. Oxidative damage to DNA comprises single and double strand breaks, DNA base modifications, abasic sites and DNA crosslinks. Cells can protect themselves against oxidative damage with antioxidants, antioxidative enzymes and repair of the lesions [30]. This balance between the formation of ROS and protective mechanisms can be destabilized in situations of excessive production of free radicals and/or of deficient antioxidant defense, resulting in oxidative stress.

In kidney disease, oxidative stress is already found in the early stages, reflected by increased levels of various biomarkers of oxidative stress [31,32]. With decline of kidney function, the oxidative stress increases [33,34]. Different sources of oxidative stress in ESRD have been identified: (1) The dialysis procedure itself causes oxidative stress, with ROS being excreted by inflammatory cells on the surface of the dialysis membranes, which depletes scavenging molecules [35,36]; (2) Indeed, a significant reduction of the glutathione based scavenging system was observed in ESRD patients, in addition to lower levels of antioxidant enzymes such as superoxide dismutase, and a vitamin C-, as well as a vitamin E-deficiency [31,37,38,39,40]; (3) The dietary restriction necessary in ESRD, with a low intake of fresh fruits and vegetables, also plays a role in the vitamin deficiency; (4) Many factors accompanying ESRD, like malnutrition or chronic volume overload, lead to a permanent state of microinflammation, which further increases ROS formation by activated neutrophils [41].

## 3. Influence of Different Dialysis Therapies on Genomic Damage in ESRD

Dialysis is applied to achieve the removal of toxic solutes and of fluid overload whereby the patient’s blood is in contact with dialysis fluid across a semi-permeable membrane. This membrane can be the natural peritoneal membrane in peritoneal dialysis or an artificial membrane in a hemodialysis filter used for extracorporeal dialysis. The driving force of the fluid and solute exchange is osmosis, hydrostatic pressure, and a concentration gradient leading to diffusion and convection [42]. Dialysis achieves a solute removal of approximately 10% of that of a healthy kidney and cannot replace the kidney’s endocrine functions. Therefore, dialysis patients are on a special diet and need supplementary medication.

The most commonly used modalities in renal replacement therapy are peritoneal dialysis (PD) and hemodialysis (HD). Recent studies recommend peritoneal dialysis as an initial therapy, when applicable, to better preserve the important residual renal function and prolong overall survival [43,44]. The benefits of PD for survival are maintained up to the third year, after which survival with PD and HD is the same, or even slightly lower in patients on PD [45,46]. HD is conventionally delivered thrice weekly for four hours either in a dialysis center or at home. An improved uremic state can be obtained by shorter interdialytic intervals in daily HD, achieving an increased removal of small solutes and lower plasma concentrations of various uremic toxins [47,48,49,50]. A further technique is hemodiafiltration (HDF), which combines the diffusion mechanism of HD with the convective component of hemofiltration. In this way, the clearance of both low and high molecular weight uremic toxins (up to 25 kDa) is improved [51,52].

### 3.1. Hemodialysis—Hemodiafiltration

A meta-analysis of 20 studies totaling 657 patients comparing convective dialysis strategies with conventional HD did not find significant differences in mortality, number of hospital admissions per year or dialysis adequacy. Due to the small number of patients included in the individual studies and the overall suboptimal quality of these studies, the convective modalities could not be evaluated definitively [53]. Not included in this analysis were the results of the large European Dialysis Outcomes and Practice Pattern Study (DOPPS), which found that patients receiving high efficiency HDF had a 35% lower mortality risk than those receiving low-flux HD [54]. Studies comparing HD and HDF observed a better clearance of middle molecules such as β2 microglobulin, leptin or complement factor D [55,56], but even small molecules like urea and phosphate were better removed by HDF [57]. This more effective removal of uremic compounds seems to be essential for the reduction of the mortality risk, since the beneficial effect persisted even after statistical adjustment for dialysis efficiency (Kt/V) [58].

HDF also had a more beneficial influence on oxidative stress parameters than did HD. Total antioxidant capacity and glutathione peroxidase activity were increased and reactive oxygen metabolites, including pentosidine, advanced oxidation protein products (AOPPs) and oxidized LDL, were decreased in patients undergoing HDF compared to HD [59,60,61,62]. In addition, the p22phox subunit of NADPH oxidase—one of the enzymes possibly partly responsible for the increased oxidative stress—was found to be less expressed in patients on HDF compared to patients on HD [62]. Last, but not least, the amount of the oxidative 8-oxodG base modification, quantified in lymphocytes after a dialysis session, was significantly lower in HDF than in HD [60], supporting the hypothesis that the HDF procedure is superior not only in eliminating molecules generating oxidative stress or produced by oxidative stress, but itself causes less oxidative stress than HD [63].

The fact that HDF better removes middle molecules, and decreases oxidative stress and also inflammation, as shown by lower C reactive protein (CRP) values of the patients [59,61], led to the assumption that patients on HDF might also show lower DNA damage. Results obtained from the comparison of the two different treatments are conflicting. One study, totaling 26 patients in the HD and 15 patients in the HDF group, did not show a difference in the DNA damage quantified with the comet assay [7]. In contrast, a yet unpublished study with 10 matched pairs of HD and HDF patients revealed less DNA damage measured by comet assay as well as a significant reduction of micronuclei in the HDF group patients (Table 1). Seven patients, who underwent a change of therapy from HD to HDF and were observed for a further eight months on HDF revealed a significant amelioration of DNA damage, as detected by comet assay, but no change in micronuclei frequency, which could be due to the rather long lifespan of lymphocytes [64]. Encompassing the observations of Vaslaki *et al.* [61], who found an improvement of many oxidative stress and inflammation parameters after switching HD patients to HDF for three months, HDF indeed might be more beneficial, concerning oxidative and genomic damage, than HD. To prove this, further studies have to be conducted analyzing these parameters.

**Table 1:** Patient characteristics, selected plasma parameters and markers of genotoxicity of matched patients on hemodialysis (red) and hemodiafiltration (blue). HD: hemodialysis; HDF: hemodiafiltration; CRP: C reactive protein; MN: micronuclei; BN: binucleated cells; CA: comet assay; 1–10: patient numbers. Methods used to obtain these data are explained in [64]. * p < 0.001 *vs.* HD; ^+^ p = 0.074; paired t-test (SPSS 18).

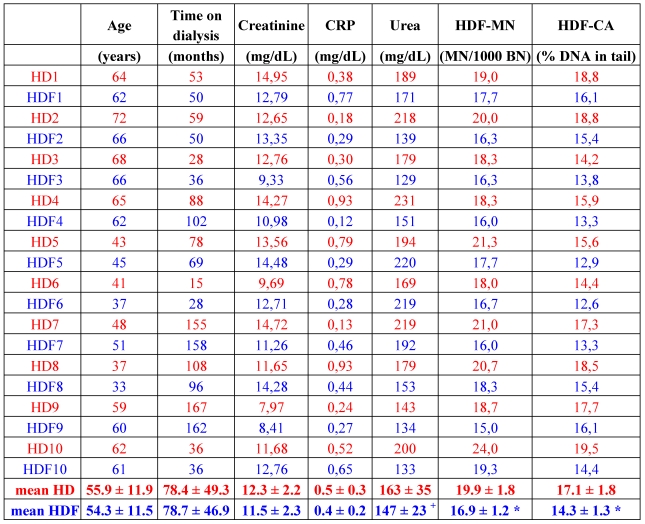

### 3.2. Hemodialysis—Daily Dialysis

Another possibility for improving dialysis therapy is to increase the time or the frequency of HD. The necessity to test such modalities has arisen out of the realization that no significant improvement of survival has been achieved despite technical advances in dialysis [65]. Survival of patients undergoing dialysis at one, two and five years of follow-up is 86%, 75% and 49%, respectively. Observations from the early 1980s, when Charra *et al.* treated patients for eight hours thrice weekly with slow flow HD and found increased survival, suggested that a dialysis session with longer duration could be more physiological and also beneficial for the patient [66,67]. Three different techniques are currently used. Short daily hemodialysis (SDHD) consists typically of 2–3.5 hours, six to seven times a week, long intermittent hemodialysis (LIHD) lasts 6–8 hours, three times a week, and nocturnal home hemodialysis (NHHD) is carried out overnight for 6–8 hours, five to seven times a week. All these dialysis modalities were studied mostly in small trials, resulting in the observation of improvements in a variety of clinical aspects and of the patients’ quality of life perception [68,69]. Additionally, a reduction in mortality between 17 to 78% was reported, as well as an increase of life expectancy of 9–15 years [70,71].

A recently published meta-analysis of 17 clinical studies analyzing the clinical effectiveness of SDHD identified possible reasons for the better outcome of patients receiving the daily treatment [72]. The clearest effect reported was the improvement of blood pressure control, resulting in a reduction of antihypertensive medication [47,73,74,75,76,77]. This is most probably achieved by a reduction of extracellular fluid excess, a lower interdialytic volume and a better control of sodium balance [72]. Erythropoetin treatment was also reduced in many patients under SDHD, explained by a better nutritional status as well as a removal of erythropoesis inhibiting uremic toxins [78,79]. Moreover, a better clearance of other uremic compounds was observed: of phosphorus [48,74,80,81,82], urea [47,48,73,74,77,82], homocysteine [83] and advanced glycation endproducts (AGEs, explained in detail below) [84]. Another beneficial aspect could be reduced inflammation as proven by lower levels of the inflammatory markers IL-6, TNF-α and CRP in SDHD patients compared to standard HD [81,85,86].

A cross-sectional study comparing patients on SDHD and standard HD therapy with regard to genomic damage, markers of microinflammation and removal of AGEs, could not detect differences in the inflammatory state [87]. Nevertheless, genomic damage assessed with the micronucleus frequency test was significantly lower under SDHD, even approaching values of the healthy control group. An explanation for this could be the better removal of uremic toxins in SDHD patients. In the same study, the improved clearance of members of the AGE group was shown, which exhibited genotoxic potential *in vitro* [24].

## 4. Effects of Vitamin Supplementation on Genomic Damage in ESRD

ESRD patients often suffer from vitamin deficiency of small water-soluble vitamins, such as vitamin C, due to a high rate of removal by dialysis and a restricted dietary intake. On the other hand, large fat-soluble vitamins, e.g., vitamin A, may be retained in the body [88]. Further reasons for the vitamin derangements in ESRD are drug interactions, which mostly affect the activity of folic acid, vitamin B_6_ and B_12_ [89]. Supplementation with vitamins in HD is therefore primarily used to compensate the deficiency. In addition, the distinct mechanisms of how the respective vitamins can ameliorate the state of health of the ESRD patients have been studied. Although the majority of investigations were conducted with supplementation of vitamin D and C, this review will now focus on studies analyzing the potential protective effects of vitamin supplementation against genomic damage in ESRD, employing vitamin E, B_1_, B_12_ and folic acid.

### 4.1. Vitamin E

While studies treating oxidative stress and inflammation with oral vitamin E produced conflicting results, the outcome of the four studies in dialysis patients concentrating on the effect of vitamin E supplementation on DNA damage unanimously show beneficial effects, which most probably can be ascribed to the antioxidative actions of vitamin E. Independent of the type of administration, oral or per vitamin E-coated dialyser membrane, DNA damage was observed to decrease [5,19,90,91]. In the two studies in which patients were given vitamin E orally, DNA damage was assessed by comet assay in one and by quantifying 8-oxodG by HPLC in the other [5,19], thereby measuring both unspecific structural DNA damage and a specific oxidative base modification. The same holds true for the two studies analyzing the impact of vitamin E-coated membranes, one applying the comet assay and the other quantifying 8-oxodG [90,91]. The latter study also included a crossover design, where patients changed the dialyser membrane type from standard cellulose to vitamin E-coated cellulose and *vice versa*. In those patients changing to the vitamin E-coated membrane, the 8-oxodG levels in leukocyte DNA significantly fell by 41% after only eight weeks, while it rose in the other group by 66% [91]. An increasing number of studies employing vitamin E-coated dialysers report favorable effects on markers of inflammation and oxidative stress, but also on blood pressure [92,93,94]. One advantage of the vitamin E-coated dialyser membrane is that the vitamin E can execute its free radical scavenging activity directly when the blood first contacts the dialyser, without activation of the inflammatory response by the membrane in the first place. Additionally, a consumption of endogenous vitamin E is prevented [91]. New vitamin E-coated dialysers based on polysulfone are being tested, which will probably be even more biocompatible than the ones coated with cellulose [92].

### 4.2. Vitamin B1

Beside oxidative stress generated by the activated immune cells, the formation of ROS can also be initiated by some uremic toxins, e.g., AGEs, which are a heterogeneous class of molecules formed by non-enzymatic reactions between reducing sugars and free amino groups of proteins, peptides, amino acids, lipids and also nucleic acids [95]. Best characterized are the protein modifications, including *N*-ε-(carboxymethyl)lysine, pentosidine and methylglyoxal-derived hydroimidazolone. The resulting adducts cannot be degraded and accumulate on long-living proteins. They are characterized by brown color, fluorescence and a tendency to polymerize. Features of the AGE-cross-linked proteins include a decreased solubility and a high resistance to proteolysis, changing the biomechanical and biochemical properties of the affected tissues [96]. AGE formation is a normal process in ageing [97]. However, in some diseases AGEs accumulate, for example in diabetes mellitus due to elevated plasma glucose levels. In ESRD, AGE accumulation results from impaired renal removal, disturbed metabolism and higher oxidative stress [98,99,100]. AGE accumulation is associated with the development of complications of diabetes (nephropathy, retinopathy and neuropathy [101]) and ESRD (dialysis-related amyloidosis, bone resorption and atherosclerosis [102,103,104]). These deleterious effects are mediated in part by the modified proteins, but also by AGE-specific receptors. The most studied receptor of AGEs, RAGE, is a multi-ligand receptor of the immunoglobulin superfamily, a signal transducing receptor modulating cellular functions [105]. Binding of AGEs to RAGE leads to oxidative stress via the induction of NADPH oxidase and to activation of proinflammatory molecules, among them NF-κB, which initiates increased expression of RAGE itself and starts a vicious circle [106,107]. These adverse effects of AGEs, together with their significantly increased plasma levels in ESRD, fulfill the criteria of uremic toxins: these compounds were classified as protein-bound uremic toxins [21].

Vitamin B_1_ (thiamine), in the form of the lipid-soluble thiamine precursor benfotiamine, was shown to inhibit the formation of AGEs [108]. Thiamine is required for the normal function of heart, muscle and nerves. It plays a key role in cellular energy metabolism, converting carbohydrates to energy [109], as the co-factor of the enzyme transketolase, which converts glyceraldehyde-3-phosphate and fructose-6-phosphate to intermediates of the pentose phosphate pathway [110]. Supplementation with benfotiamine in diabetic patients demonstrated a significant relief in neuropathic pain and a marked improvement in vibration perception thresholds [111]. Vitamin B_1_ has been shown to be decreased in many diabetic patients due to enhanced urinary excretion and malabsorption [111]. This deficiency manifests itself in reduced transketolase activities compared to healthy subjects [112]. Low transketolase is connected to neuropathies observed in approximately 25% of uremic patients and probably contributes to the unexplained cases of encephalopathy in the dialysis population [113,114,115].

Strikingly, although thiamine deficiency in dialysis patients was already observed in the early 1980s [116] and the hypothesis of a role of transketolase deficiency in uremic neuropathy exists since the 1970s [113], to date no clinical studies have analyzed the effect of thiamine supplementation on neurological problems in dialysis patients. By contrast, first clinical trials of thiamine in diabetic patients with neuropathies were conducted as early as 1992 and reported improvement of the symptoms [117]. Pharmacokinetic studies in dialysis patients were carried out, revealing the superiority of the bioavailability of benfotiamine *versus* thiamine [118].

Benfotiamine, therefore, was used in the first studies analyzing the impact of vitamin B1 supplementation on genomic damage of HD patients [119]. In two small consecutive prospective studies totaling 30 treated patients, the administration of 450–600 mg per day of benfotiamine for four months successfully decreased the micronucleus frequency of peripheral lymphocytes. The number of micronuclei was inversely correlated to the erythrocyte transketolase activity. Surprisingly, no effect of benfotiamine on plasma content of fluorescent AGEs could be detected. Since an intrinsic antioxidative capacity of benfotiamine unrelated to its anti-AGE effects was observed *in vitro* as well as *in vivo* [120,121], and the antioxidative plasma capacity of the benfotiamine-treated patients increased, the reduction of genomic damage can be explained with this property of benfotiamine. Possibly longer observation periods in humans are needed to reliably detect a lowering of plasma AGEs.

### 4.3. Folic Acid in Combination with Vitamin B12

Another uremic toxin whose formation can probably be influenced by supplementation with vitamins is homocysteine. Hyperhomocysteinemia was detected in approximately 85% of ESRD patients and persists throughout dialysis and even after renal transplantation [122]. Homocysteine is a normal intermediate in methionine metabolism, usually recycled to methionine with the involvement of folic acid and vitamin B_12_ [123]. Methionine is used for protein synthesis and, after conversion to *S*-adenosylmethionine, is an important methyl donor for transmethylation reactions, needed for example for DNA cytosine-methylation. Inhibition of DNA methyltransferases by *S*-adenosylhomocysteine was observed in individuals with increased homocysteine plasma levels, accompanied by hypomethylated DNA [124]. Insufficient DNA methylation renders the DNA less stable [125], possibly leading to genomic damage. Indeed, a correlation between plasma homocysteine levels and the number of micronuclei was found in humans [126].

Clinical studies analyzing the efficacy of folic acid alone or in combination with vitamin B_12_ (sometimes further combined with vitamin B_6_) on homocysteine levels in dialysis patients report inconsistent results. The plasma level of homocysteine was reduced markedly in only a few of the recently conducted trials [127,128], while there were only small changes in others [129,130]. The primary outcomes of the larger studies, all-cause mortality, cardiovascular events or cognitive function, were not ameliorated at all by the vitamin supplementations [127,131,132,133].

To date, the only study to explore the effect of supplementation with folic acid and vitamin B_12_ on genomic damage in dialysis patients observed a significant decline in plasma homocysteine levels, but no corresponding changes in the percentage of DNA methylation [134]. Nevertheless, genomic damage, assessed as micronucleus frequency, decreased in a therapy-length dependent manner. A possible antioxidant capacity of the supplemented vitamins could account for this beneficial effect.

## 5. Further Uremic Toxins with Genotoxic Properties

Apart from the above described uremic toxins with genotoxic effects, advanced glycation endproducts and homocysteine, further substances classified as uremic toxins show DNA damaging effects (Table 2). This was confirmed primarily by the comet assay, but also by detection of chromosome breaks and formation of the oxidative base modification 8-oxodG. It can be assumed that these molecules contribute to the increased genomic damage observed in dialysis patients. Since the uremic syndrome is characterized by accumulation of multiple compounds, synergistic actions of these compounds, amplifying their genotoxic potential, cannot be ruled out. Identification of additional uremic toxins and intensified research uncovering their toxicological properties will lead to an expansion of Table 2.

**Table 2:** Examples of uremic toxins [21,22], with published evidence of DNA-damaging potential.

Uremic toxin	DNA damage	References
Angiotensin II	8-oxodG, micronuclei, double strand breaks, abasic sites	[135,136]
Hydroquinone	DNA strand breaks, micronuclei, 8-oxodG	[137]
Indoxyl sulfate	DNA strand breaks	[120]
Leptin	DNA strand breaks	[6]
Methylglyoxal	DNA strand breaks, 8-oxodG	[24,138]
*N*-ε-(carboxymethyl)lysine	DNA strand breaks	[24]
*N*-methyl-2-pyridone-5-carboxamide	DNA strand breaks	[139]
Nitrosodimethylamine	DNA strand breaks, micronuclei, known rat-liver carcinogen	[140,141,142]
Thiocyanate	DNA strand breaks	[143]
TNF-α	8-oxodG, DNA strand breaks	[25,144]

## 6. Conclusions

The possible modulation of genomic damage in ESRD by more intensive HD techniques more effectively removing uremic toxins and by supplementation with substances counteracting adverse effects of uremic toxins further supports the presumption that these compounds are mainly responsible for the formation of the DNA damage. The ability of a uremic toxin to induce the generation of oxidative stress seems to be a prerequisite for the formation of DNA damage. In addition to improving the overall condition of dialysis patients, HD techniques ensuring a more effective removal of uremic toxins, could also reduce the accumulation of genomic damage, possibly preventing the complication “malignancy” in dialysis patients.
